# Flow cytometry-based viability staining: an at-line tool for bioprocess monitoring of *Sulfolobus acidocaldarius*

**DOI:** 10.1186/s13568-022-01447-1

**Published:** 2022-08-10

**Authors:** Kerstin Rastädter, Andrea Tramontano, David J. Wurm, Oliver Spadiut, Julian Quehenberger

**Affiliations:** 1 Research Division Biochemical Engineering, Faculty of Technical Chemistry, Institute of Chemical, Environmental and Bioscience Engineering, TU Wien, 1060 Vienna, Austria; 2 Department of Functional and Evolutionary Ecology, Archaea Biology and Ecogenomics Unit, University of Vienna, 1030 Vienna, Austria; 3 NovoArc GmbH, 1060 Vienna, Austria

**Keywords:** Flow cytometry, *Sulfolobus acidocaldarius*, Fluorescent dyes, Viability, Live/dead staining

## Abstract

Determination of the viability, ratio of dead and live cell populations, of *Sulfolobus acidocaldarius* is still being done by tedious and material-intensive plating assays that can only provide time-lagged results. Although *S. acidocaldarius,* an extremophilic Archaeon thriving at 75 °C and pH 3.0, and related species harbor great potential for the exploitation as production hosts and biocatalysts in biotechnological applications, no industrial processes have been established yet. One hindrance is that during development and scaling of industrial bioprocesses timely monitoring of the impact of process parameters on the cultivated organism is crucial—a task that cannot be fulfilled by traditional plating assays. As alternative, flow cytometry (FCM) promises a fast and reliable method for viability assessment via the use of fluorescent dyes. In this study, commercially available fluorescent dyes applicable in *S. acidocaldarius* were identified. The dyes, fluorescein diacetate and concanavalin A conjugated with rhodamine, were discovered to be suitable for viability determination via FCM. For showing the applicability of the developed at-line tool for bioprocess monitoring, a chemostat cultivation on a defined growth medium at 75 °C, pH 3.0 was conducted. Over the timeframe of 800 h, this developed FCM method was compared to the plating assay by monitoring the change in viability upon controlled pH shifts. Both methods detected an impact on the viability at pH values of 2.0 and 1.5 when compared to pH 3.0. A logarithmic relationship between the viability observed via plating assay and via FCM was observed.

**Supplementary Information:**

The online version contains supplementary material available at 10.1186/s13568-022-01447-1.

## Key points

- Development of a flow cytometry (FCM) method for viability determination of S. acidocaldarius using the fluorescent dyes fluorescein diacetate and concanavalin A conjugated with rhodamine.
- Applicability of the developed method was shown via viability monitoring during a continuous cultivation with triggered pH shifts.
- A logarithmic trend was observed between the developed FCM method and the state-of-the-art method, plating assay.

**Supplementary Information:**

The online version contains supplementary material available at 10.1186/s13568-022-01447-1.

## Introduction

Flow cytometry (FCM) has shown to be a powerful technique for analyzing a broad spectrum of cell parameters on a single cell level (Díaz et al. 2010; Adan et al. 2017; Maciorowski et al. 2017; Robinson 2018). It has been used for decades for the characterization of organisms from all domains of life (Scheper et al. 1987; Forment et al. 2012; Vees et al. 2020) and most applications of FCM are based upon fluorescence labelling. An important application of FCM in biotechnology is the determination of live and dead cells in a cultivation which is a key parameter for bioprocess development and control (Rieseberg et al. 2001). Successful applications of FCM have been published for the well-known mesophilic industrial production host *Escherichia coli* (Wurm et al. 2017; Kopp et al. 2020) and for *Pichia pastoris* (Hohenblum et al. 2003), highlighting how bioprocess development can benefit from FCM-based viability monitoring. These studies showed that measuring the ratio of dead cells to the total cell count can be used for monitoring the effect of process parameters (Wurm et al. 2017) and on the other hand the effect of different cultivation strategies (Hohenblum et al. 2003; Kopp et al. 2020) on process performance.

In recent years, growing interest in more exotic biotechnological production hosts can be observed. Members of the order Sulfolobales have been proposed as potential key organisms for future bioprocesses, harboring unique metabolic pathways and many valuable products such as extremozymes, highly stable lipids in their cell membrane as well as antibiotic complexes (Quehenberger et al. 2017; Rastädter et al. 2020). *Sulfolobus acidocaldarius* has emerged as an important model organism for the phylum Crenarchaeota due to its stable and fully sequenced genome (Chen et al. 2005) and its well-developed toolbox for genetic engineering (Wagner et al. 2012; Peng et al. 2017). It was only recently considered as an organism of high potential in biotechnological applications (Zeldes et al. 2015; Quehenberger et al. 2017; Schocke et al. 2019). Hence, physiological strain characterization is only in its beginnings (Rastädter et al. 2021) and no at-line methods for viability determination for fast identification of critical process parameters exist for *S. acidocaldarius*. Although, in the literature a number of fluorescence based, culture-independent detection methods for Sulfolobales species can be found (Brock et al. 1972; Bernander and Poplawski 1997; Hjort and Bernander 1999; Bernander 2007; Han et al. 2017), these methods do not assess the cell viability. Therefore, the current state-of-the-art method for determining the viability of a population is still via plating assay (Lindström and Sehlin 1989; Han et al. 2017). The outcome is time-delayed and depends on various factors for accurate results, such as correct dilution of cell suspension prior to plating, correct counting as well as subjective definition of a colony.

Generally, a cell is determined viable if it is capable of reproduction. While this property is directly assessed by plating assays that yield colony forming units (CFUs) as a response, for fast at-line estimation of viability it is necessary to find proxies that can be determined independently of time-consuming incubation periods. Since metabolic activity is required for cell division, measuring the activity of representative housekeeping enzymes, like esterases, can be used as an indirect, though at-line way for determining the viability (Nyström 2001; Cangelosi and Meschke 2014). Following this principle, FCM analysis combined with fluorescent dyes, such as fluorescein diacetate (FDA) (Xiao et al. 2011), can evaluate the cell’s metabolic activity, thereby allowing the estimation of the viability of a microbial population on a cellular level.

This study aims to develop an at-line method for bioprocess monitoring of the viability of *S. acidocaldarius* by using FCM in combination with suitable fluorescent dyes as a tool for efficient bioprocess monitoring. This method is then compared to the state-of-the-art method, plating assay, during a continuous bioreactor cultivation with triggered changes in viability via shifts in the pH value.

## Material and methods

### Fluorescence microscopy

A Leica DMI 8 fluorescence microscope (Leica Microsystems, Germany) equipped with a mercury light source was used for screening of fluorescence dyes. Three filter configurations were available: Filter 1, excitation (ex.) 532–558 nm/emission (em.) 570–640 nm); Filter 2, ex. 450–490 nm / em. 500–550 nm; Filter 3, ex. 600–660 nm / em. 662–738 nm. Undiluted cell suspensions were applied on glass slides and after addition of fluorescent dyes samples were incubated in the dark over a range of 5–50 min before investigation with the microscope.

### Screening of fluorescent dyes

Eight different fluorescent dyes were investigated regarding their suitability for cell staining of *S. acidocaldarius* (Table 1).

**Table 1:** Overview of the investigated fluorescent dyes

Dye	Ex. λ_max_^1^ [nm]	Em. λ_max_^2^ [nm]	Fluorescence color	Permeability*	Mode of interaction	For detection of
Acridine orange (AO)	500	526	green	permeable	DNA/RNA	viable and non-viable cells
SYTO™ 9	485	500	green	permeable	DNA/RNA	viable and non-viable cells
RH414	532	716	red	permeable	cell membrane	viable and non-viable cells
Concanavalin A-rhodamine	545	570	red	impermeable	cell membrane	viable and non-viable cells
Fluorescein diacetate (FDA)	485	520	green	permeable	enzymatic fluorophore generation	viable cells
DiBAC_4_(3)	493	516	green	impermeable	positively charged or hydrophobic regions	non-viable cells
Propidium iodide (PI)	535	617	red	impermeable	DNA/RNA	non-viable cells
7-AAD	546	647	red	impermeable	DNA, G-C rich regions, RNA	non-viable cells

Acridine orange (AO) was obtained from Carl Roth (Germany). SYTO™ 9 and 7-Aminoactinomycin D (7-AAD) were purchased from Thermo Fisher Scientific (USA). RH414 [N-(3-Triethylammoniumpropyl)-4-(4-(4-(diethylamino)phenyl)butadienyl) pyridinium dibromide] as well as DiBAC_4_(3) [Bis- (1,3-dibutylbarbituric acid) trimethine oxonol] were obtained from AnaSpec (USA). Concanvalin A—rhodamine was supplied by Vector Laboratories (USA) while fluorescein diacetate (FDA) and propidium iodide (PI) was purchased from Sigma Aldrich (USA)

^1^Ex λ_max_: maximum excitation wavelength

^2^Em λ_max_: maximum emission wavelength

*here permeability describes the capability of a dye to enter intact (uncompromised) cells

### Strain and bioreactor setup

*Sulfolobus acidocaldarius* DSM 639*,* obtained at German Collection of Microorganisms and Cell Cultures (DSMZ, Germany), was grown continuously in a 2 L Biostat A-plus bioreactor (Sartorius, Germany). The culture was stirred at 350 rpm, supplied with 0.23 vvm (0.45 L/h) pressurized air and kept at a constant temperature of 75 °C. The pH was measured with an Easyferm Plus Electrode (Hamilton, USA) and the dissolved oxygen (dO_2_) was monitored by a VisiFerm DO225 probe (Hamilton, USA). pH was adjusted by automatic addition of 4.8% (v/v) H_2_SO_4_. CO_2_ and O_2_ concentrations in the exhaust gas were measured using a gas analyzing unit (Müller Systems AG, Switzerland). The cultivation was monitored and controlled using the Lucullus process control system (SecureCell, Switzerland). The batch phase was started with an initial OD_600_ of 0.275 in 1.5 L of Vienna Defined (VD) Medium (Quehenberger et al. 2019) with modified concentrations of carbon sources (2 g/L monosodium glutamate (MSG), 1 g/L d-glucose). During the fed-batch phase an exponential feed was applied, starting with 14.8 g/h and a growth rate of 0.035 h^−1^. After reaching 2 L working volume a dilution rate of 0.03 h^−1^ was established and the chemostat phase was started. Feed used during fed-batch and chemostat phases contained a 5-times concentrated VD Medium with modified carbon source concentrations (9.5 g/L MSG, 4.5 g/L d-glucose and 0.5 g/L NZ-amine (Sigma, USA, a protein hydrolysate containing all 20 amino acids). For maintaining a constant volume during chemostat cultivation, cell broth was pumped out of the reactor via a bleed tube at a fixed height.

Biomass for the following viability experiments was harvested during the chemostat phase. To trigger changes in viability, the pH was changed from the standard value of 3.0 to 2.0 and 1.5, respectively.

### Biomass determination

Optical density (OD_600_) was determined via a spectrophotometer (ONDA V-10 PLUS, XS instruments, Italy) at 600 nm against a blank of deionized water.

### Plating assay: viability determination

Since plating is the state-of-the-art method for viability determination with *S. acidocaldarius* (Lindström and Sehlin 1989; Han et al. 2017), this method was used as benchmark in the present study. The generated response of the plating method are CFUs, consequently this method assesses the capability of replication. 400 mL 1.2% gelrite and 400 mL 2× concentrated brock medium (Brock et al. 1972; Quehenberger et al. 2019) supplemented with 0.4 g CaCl_2_ and 0.76 g MgCl_2_ were prepared aseptically. Glucose with a final concentration of 2 g/L and 1 g/L g NZ-Amine served as the carbon sources. After their preparation the two solutions were mixed and the pH was set to 3.0 with 4.8% H_2_SO_4_ prior to pouring á 20 mL into 94 × 16 mm petri dishes (Greiner Bio-One, Austria). Depending on the OD_600_ and on the expected CFUs the cell broth after sampling was diluted 10^–4^ to 5·10^–6^ with VD Medium without carbon sources. 50 µL of this suspension was pipetted onto the plate and dispersed. After 6 days at 75 °C incubation, the CFUs were determined. Each sampling point was plated in four replicates. The specific viability, V_plating_ [CFU/ml/OD_600_], was calculated as follows: ${V}_{plating}= \frac{CFU*dilution factor}{O{D}_{600}*{Volume}_{plated}}$.

### Flow cytometry: viability determination

At each sampling point, 300 µL of cell broth were centrifuged in a 2 mL Eppendorf tube at 10,000×*g*, 4 °C and for 10 min. The cell pellet was washed twice and resuspended with 0.2 µm-filtered 10 mM phosphate buffered saline (PBS), pH 5.5. In between the washing steps, a five-minute centrifugation step at 20,000×*g* and 4 °C was carried out. Then, 600 µL of PBS buffer were added to the 300 µL washed cell suspension to yield a 1:3 dilution. 4.5 µL fluorescein diacetate (FDA, 5 g/L in acetone, Sigma- Aldrich, USA) were added to the obtained 900 µL. The sample was then incubated for 10 min at 37 °C in a thermoblock. After incubation, another centrifugation step at 20,000×*g*, 4 °C and for 5 min occurred to reduce background fluorescence caused by released fluorescein in the supernatant. The cell pellet was resuspended in 900 µL PBS buffer.

After FDA staining, the sample was diluted 1:3000 with PBS buffer to a final OD of 0.002 to 0.003. 50 µL concanavalin A (ConA)-rhodamine (5 g/L, Vector Laboratories, USA) were centrifuged prior to use to remove any possible protein aggregates which may form during storage. Shortly before measuring, 1 µL of ConA-rhodamine was added to 1 mL diluted FDA-stained cell suspension. The measurement was performed using a Cyflow Cube 8 flow cytometer (Sysmex, Germany), equipped with a 488 nm blue and 532 nm green laser. Emission spectra were obtained with fluorescence channels FL1, 536/40 nm bandpass, and FL4, 610/30 nm bandpass filter. Additionally, a forward scatter (FSC) and side scatter (SSC) detection was available. As all cells are stained by ConA-rhodamine, the emission spectra were acquired by using the FL4 as a trigger parameter. Opensource software FCSalyzer (Mostböck) was used for data visualization. The viability identified via the FCM was termed V_FCM_ and was determined as follows: ${V}_{FCM}=\frac{viable cells}{all cells}$.

To obtain *non-viable* cells, the cells were killed via suffocation at high temperature. This was done by pouring 1 mL of cell suspension in a 2 mL Eppendorf tube which was then sealed with parafilm. The tube was put in a 100 mL Erlenmeyer flask filled with water and closed off with aluminum foil. The flask was transferred to a 75 °C-oil bath and was incubated over night while shaking. By doing this procedure, the remaining oxygen in the cell suspension is consumed resulting in depletion of ATP. Eventually, this leads to acidification of the cytosolic pH, which can no longer be maintained at the physiological value of 6.5 due to inactivity of proton pumps and simultaneous influx of H^+^ ions form the surrounding medium. The respective *non-viable* cell suspension sample was then treated like mentioned above. For calibration purposes and to determine the sensitivity of the method, the *non-viable* and *viable* samples after FDA staining were mixed in the respective ratios.

## Results

### Method development

#### Screening of fluorescence dyes

Compared to mammalian cells and Bacteria, for Archaea only a limited number of fluorescent dyes are commercially available (Johnson and Spence 2010). Nevertheless, SYTO 9 and acridine orange (AO) have already been tested for the use in Archaea and have been reported to successfully stain *Sulfolobus* cells (Brock et al. 1972; Leuko et al. 2004) and detect dead/all cells in haloarchaea (Leuko et al. 2004). In this study, 8 fluorescent dyes, listed in Table 1, were tested regarding their applicability for viability determination of *S. acidocaldarius*. Only 5 of the 8 investigated dyes led to fluorescent cells under the fluorescence microscope: AO, FDA, SYTO 9, ConA-rhodamine and propidium iodide. Since the viability determination of a population via the fluorescence microscope is time consuming and highly operator dependent, and hence would defeat the purpose of finding an at-line monitoring tool, a fluorescence microscope-based viability assay was not pursued within this study. Instead, an FCM-based approach was chosen as this method generates results within minutes after the sample is prepared.

### Cell staining with FCM

FCM is described as a reproducible method for acquiring optical and fluorescence information of cultures on a single cell level (Rieseberg et al. 2001; Díaz et al. 2010). Generally, to determine viability of a cell culture via FCM, at least one fluorescent stain that highlights a specific characteristic such as *viable* or *non-viable* is needed. Cells can either be distinguished from the background noise due to size in the forward scatter or by using an additional fluorescent dye that specifically stains all cells. By combining these characteristics, two populations can be identified. Out of the five dyes that stained cells in the fluorescence microscope, only AO, FDA and ConA-rhodamine also showed a fluorescent signal when investigated with the flow cytometer. AO permeates cells regardless of their membrane integrity and subsequently leads to fluorescence emission when bound to intracellular nucleic acid (Martens-Habbena and Sass 2006). FDA staining is based on development of fluorescence upon cleavage of the FDA molecule by cellular esterases. The produced fluorescein accumulates inside the cell, resulting in green fluorescence when excited. Consequently, compromised cells with a reduced amount of esterase activity exhibit a reduced fluorescence signal compared to healthy cells (Jones and Senft 1985). Although, complementary staining properties are given in case of AO (stains all cells) and FDA (only living cells) it is not feasible to use the two dyes simultaneously in a single assay, since both dyes have similar emissions maxima (526 vs. 520 nm) and consequently, due to spectral overlap their signals cannot be discriminated. ConA-rhodamine was used instead, which binds to α-linked mannose present in the core oligosaccharides in the membrane glycoproteins (Fontaniella et al. 2004). In Sulfolobales mannose is present in the glycoslylated S-layer protein SlaA and flagellin FlaB (Elferink et al. 2001; Peyfoon et al. 2010; Zolghadr et al. 2010; Meyer et al. 2011). ConA conjugated with rhodamine (stains all cells), which has an emissions maximum of 575 nm, can be used in combination with FDA. Hence, the rhodamine emission in FL4 (610/30 nm bandpass) determined all cells, while the fluorescence signal originating from FDA hydrolysis in FL1 (236/40 nm bandpass) identified the *viable* cells. Fluorescence microscope images, showing cells stained with FDA and ConA-rhodamine and an overlay of both figures can be seen in the Additional file 1. Thereby it is shown that all cells exhibit red fluorescence resulting from ConA-rhodamine (Additional file 1: Figure S1B). However, *viable* cells additionally exhibit green fluorescent from FDA staining (Additional file 1: Figure S1A, C). In this paper, for better readability metabolic inactive/active cells according to the FCM are described as *non-viable*/*viable*.

### Determination of viability via FCM (VFCM)

**Fig. 1:**
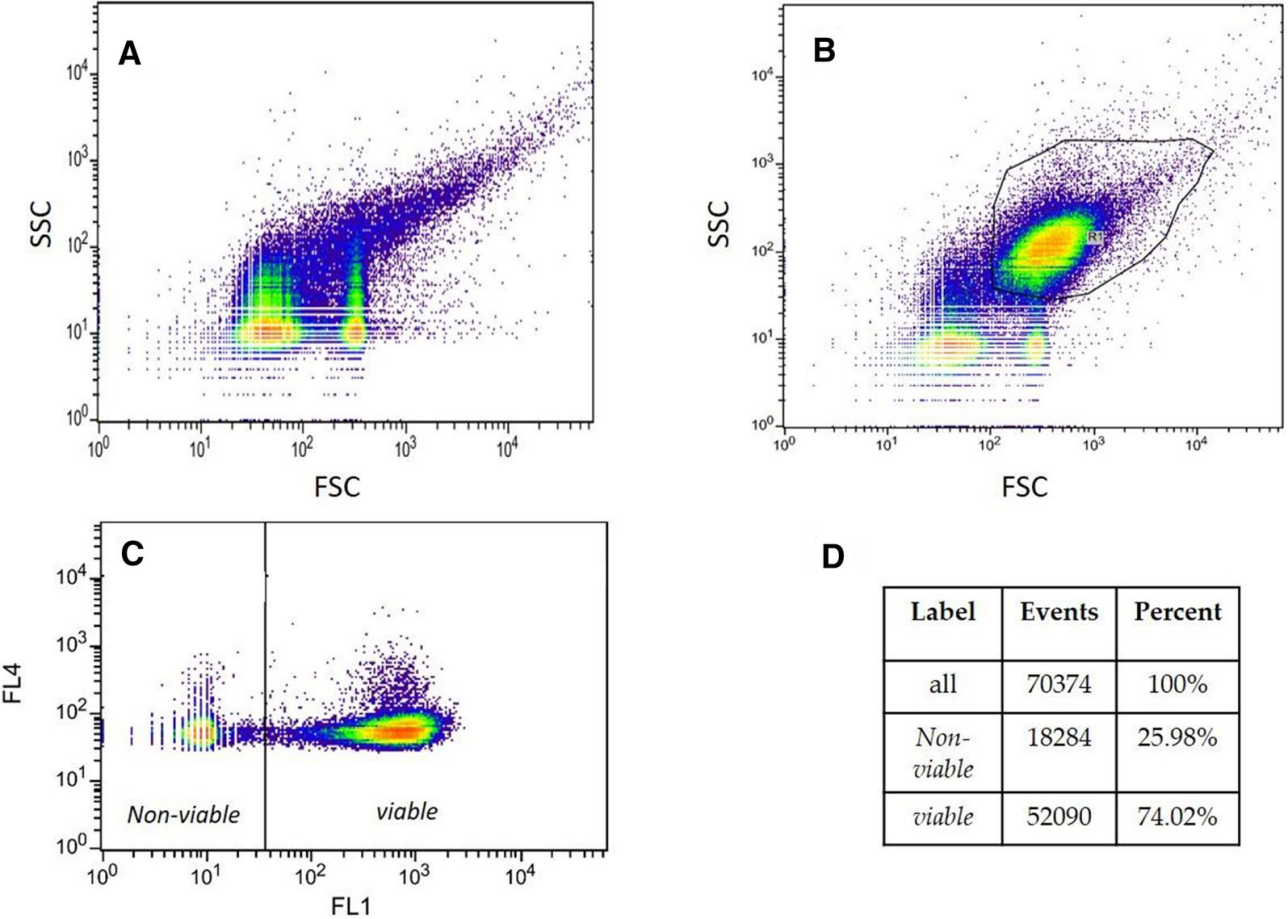
Gate definition for viability evaluation of *Sulfolobus acidocaldarius*. **A** density plot of side scatter versus forward scatter for ConA-rhodamine in PBS buffer, showing the background, color code: red-high to purple-low; **B** density plot of side scatter versus forward scatter for cells stained with FDA and ConA-rhodamine, showing the cell gate; **C** density plot of FL1 (536/40 nm bandpass) versus FL4 (610/30 nm bandpass) of cells gated in **B**; **D** statistics of FL1 vs. FL4 shown in **C**

By setting the trigger parameter to FL4, only particles that are ConA-rhondamine stained were recorded, thereby reducing particle events. While PBS buffer spiked with ConA-rhodamine still generated background noise, caused by unspecific fluorescence and aggregates (Fig. 1A), the cell population (gated in Fig. 1B) could be clearly distinguished from this background via the FSC and SSC plot due to the cell size and form. The cell gate was then used for determining the viability in FL1 (showing FDA stained cells) versus FL4 plot (Fig. 1C). On the left side with low FL1 intensity the *non-viable* cells can be seen and on the right side the *viable* cells.

### Sensitivity analysis and comparison with state-of-the-art

**Fig. 2:**
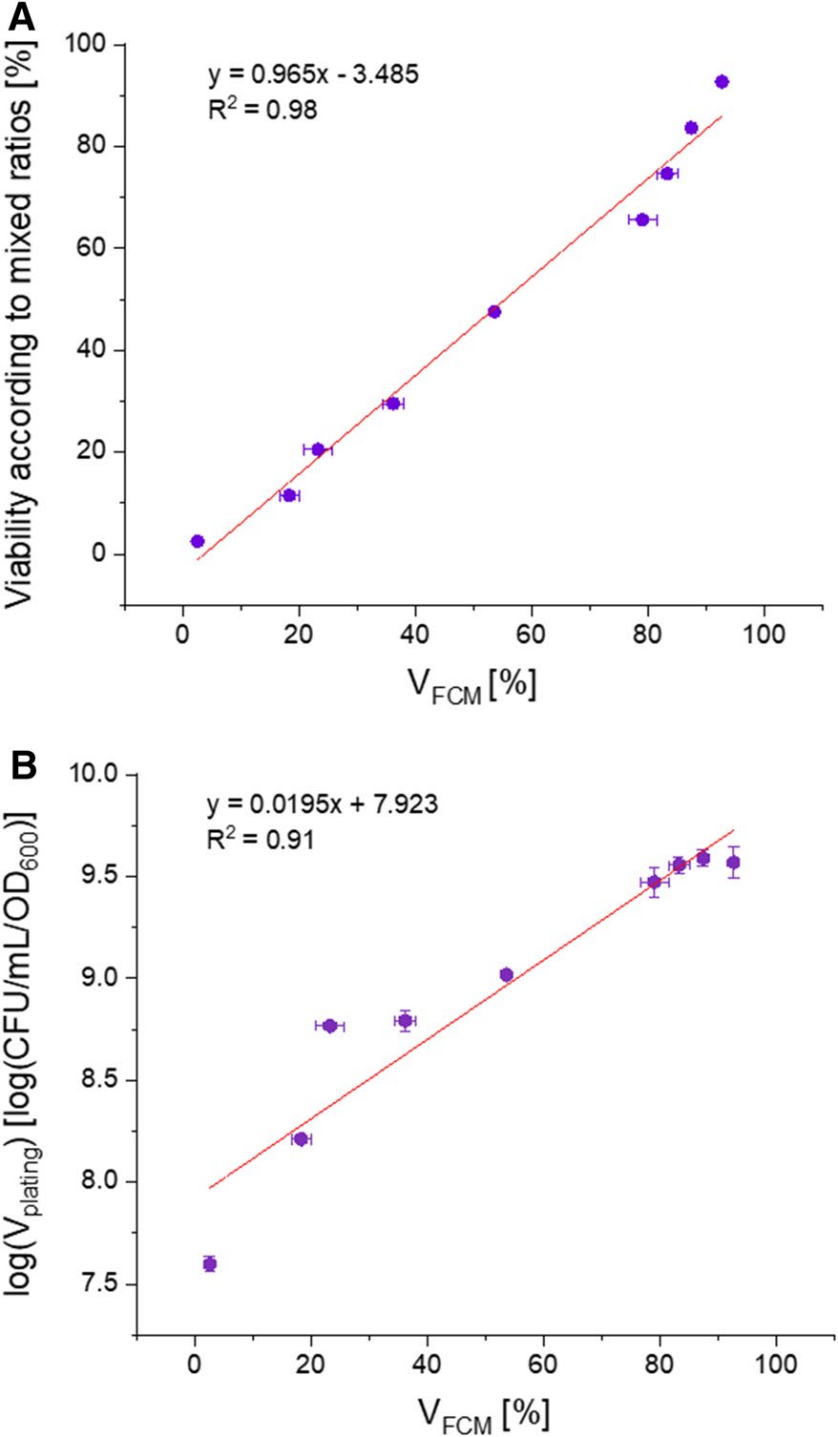
**A** Sensitivity analysis of viability according to mixed ratios [%] vs. V_FCM_ [%]. Viability according to mixed ratios [%] were obtained by mixing different ratios of *non-viable* and *viable* cell populations. V_FCM_ cells [%] were measured by the flow cytometer and evaluated according to Fig. 1. **B** Comparison of state-of-the-art method log[V_plating_ (CFU/mL/OD_600_)] versus V_FCM_ [%]

Cells were killed by O_2_ depletion and then mixed with *viable* cells in certain ratios. Since the sample of *viable* cells already contained around 7% *non-viable* cells, the amount was subtracted from the calculated percentage of live cells based on the mixed ratios. The trend between measured *viable* cells and calculated *viable* cells (according to the mixed ratios) in the measured populations shows a linear correlation between the determined viability (V_FCM_) and the percentage of calculated *viable* cells in a sample over the range of 2.5–92.7% (Fig. 2A). For comparison with the state-of-the-art method plating (V_FCM_ vs. V_plating_), the same mixtures of *viable* and *non-viable* cells were also cultivated on gelrite plates and the CFUs were determined 6 days later. The relationship between the CFUs of the plated *viable/non-viable* mixtures and the V_FCM_ shows a logarithmic trend (Fig. 2B).

### Applicability test of FCM method

**Fig. 3:**
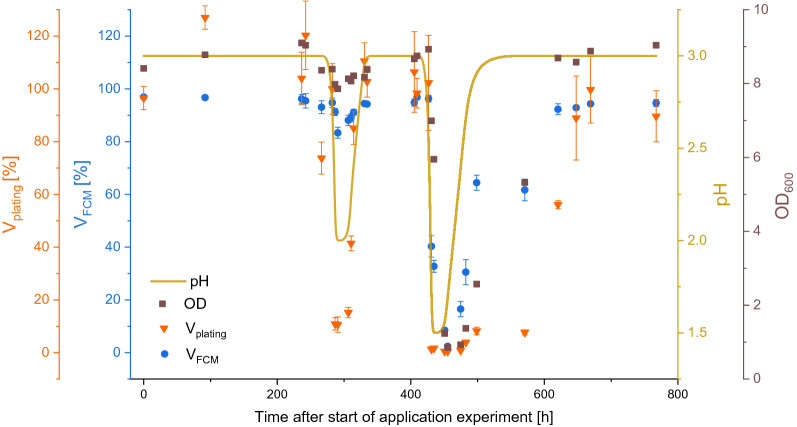
Comparison of viability measurements of *Sulfolobus acidocaldarius* in response to the shift in pH value, observed over time [h]. The viability was determined by flow cytometry (V_FCM_ [%]) and by plating assay (V_plating_ [%]). Both pH shifts from 3.0 to 2.0 and to 1.5, respectively caused a drop in both viability-determining methods as well as in OD_600_. In this case, V_plating_ measured at the beginning of the experiment was set to 100%, and for determining V_plating_ [%], the CFUs of each sample were then divided by this initial value

**Fig. 4:**
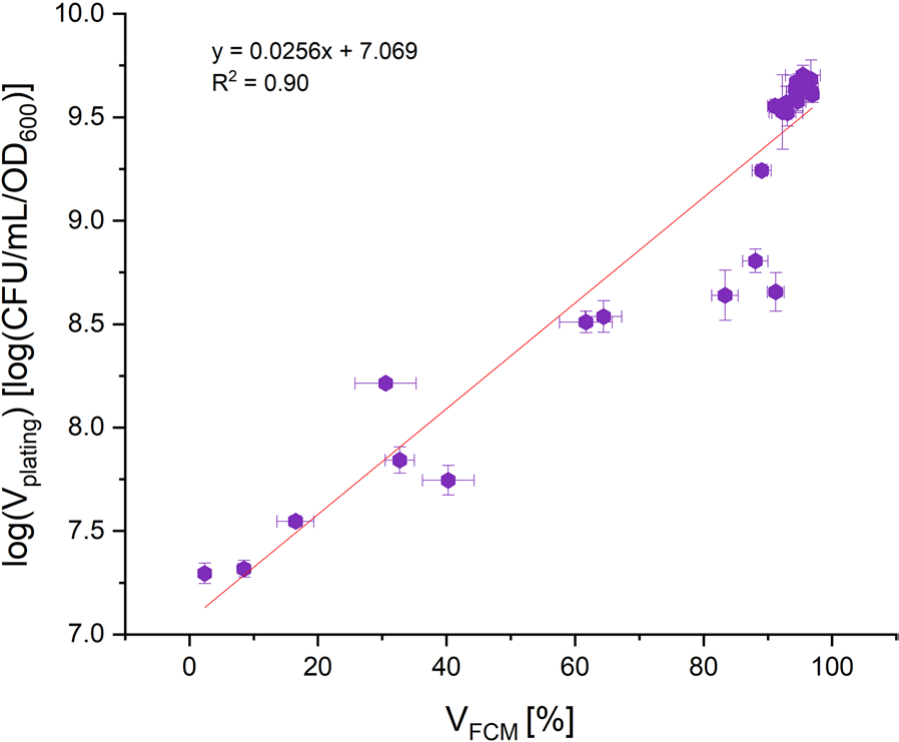
Logarithmic trend of V_FCM_ [%] versus log (V_plating_) with a correlation factor of R^2^ = 0.90

For showing the applicability of the developed FCM method, continuously cultivated cells were subjected to stress induced by pH changes. The viability was monitored by FCM (V_FCM_) and plating (V_plating_) throughout these pH changes. V_FCM_ and V_plating_ decreased in response to the shift towards more acidic conditions (Fig. 3). This effect was less prominent after the pH value was changed from the pH optimum of 3.0 (Rastädter et al. 2021) to 2.0 and more severe when a pH value of 1.5 was applied. After shifting back to the pH optimum of 3.0, the cells recovered in both cases and V_FCM_ and V_plating_ resumed the same values as before the pH shift. The relation between V_FCM_ and V_CFU_ is shown in Fig. 4. Similar to the relationship in the sensitivity analysis where cells were killed via oxygen depletion (Fig. 2B) also here a logarithmic relationship was observed as the pH shifts apparently impacted V_plating_ more severely than V_FCM_.

## Discussion

FCM methods applied to other organism have shown to be a robust tool for getting an insight into the viability of a cell population (Xiao et al. 2006; Wurm et al. 2017; Vees et al. 2020). Additionally, it could be shown that the impact of process parameters such as feeding strategy (Xiao et al. 2006; Vees et al. 2020) and cultivation temperature (Wurm et al. 2017) can be monitored.

Despite significant background signals for all cells during ConA-rhodamine staining (Fig. 1A), a clear differentiation between the background and cells can be made when gating based on forward/side scatter, thereby adding information on characteristic shape/size of the cells (Fig. 1B). The resulting trend in the sensitivity analysis (Fig. 2A) showed a linear range between 2.45 and 92.72% of V_FCM_. A logarithmic correlation between state-of-the-art method plating assay and the developed FCM method was observed (Fig. 2B). A possible explanation for the logarithmic nature of the correlation could be that these methods investigate viability differently. Plating on gelrite plates [CFU/mL/OD_600_] and FDA/ConA-rhodamine staining combined with flow cytometry are based on different mechanisms to determine cell viability. With one method, the number of proliferable cells upon transfer to a different growth environment (gelrite plates) is obtained, while the other method examines the metabolic activity of each cell as an at-line measurement (Cangelosi and Meschke 2014). Apparently, cells harvested during the shift to pH 2.0 were already significantly impaired in their ability to form colonies in the gelrite plate assay. This circumstance was neither mirrored in the limited drop of OD_600_ nor in the moderate reduction of V_FCM_ during this shift to pH 2.0, while all three parameters (V_plating_, V_FCM_, OD_600_) dropped significantly when the pH was reduced even further to a value of 1.5.

Although it is the current standard method for viability determination (Lindström and Sehlin 1989; Han et al. 2017), the plating assay applied in this paper harbors limitations that can explain possible divergence in the viability when compared to V_FCM_. First, cells could retain metabolic activity while being unable to divide (Rollins and Colwell 1986; Nyström 2001; Cangelosi and Meschke 2014). The inability of proliferation despite metabolic activity could explain the drastic changes in V_plating_ upon pH value changes while the V_FCM_ of the culture only moderately decreased (Fig. 3). This is also mirrored in the logarithmic relationship between V_FCM_ and V_plating_ (Figs. 2B and 4). Suffocation (Fig. 2B) as well as a pH drop (Fig. 4) lead to a decrease of cytosolic pH as a result of too low proton pump activity (Anemüller et al. 1985; Gleissner et al. 1997; Baker-Austin and Dopson 2007). Due to the cytosolic pH decrease, adaption to this new condition is necessary and apparently at the beginning of this process the cells are metabolically active, but reproduction is stalled (Fig. 3). Secondly, in the plating assay the standard medium pH value is 3.0 (Wagner et al. 2012; Bischof et al. 2019). However, the pH value of the preceding cultivation conditions might differ from this pH optimum of 3.0. Hence, the cells have to overcome another pH adaption in order to grow, which could cause the lower viability in comparison to V_FCM_.

The time-delay for obtaining the results after sampling is still the biggest limitation, making plating assay-based bioprocess monitoring unfeasible. Additionally, wrong dilutions of the cell suspension prior to plating could lead either to too many colonies or too little to count, which both causes inaccurate results—a fatal error that cannot be corrected, since the actual sampling time already lies at least 6 days in the past. Incorrect dilutions during a FCM measurement can easily be detected and a re-run of the sample is possible immediately. In the evaluation process of plating, the timing for counting the colonies on the plate is of essence. If it is done too late two colonies can merge and appear as one, on the other hand small colonies can be overlooked in an early evaluation.

The collective disadvantages of plating show the need for a robust and at-line FCM method with high statistical significance due to high sample size and high event numbers to determine the viability in a cell population. The developed FCM method harbors many applications in the rising field of *S. acidocaldarius* bioprocessing. It can be used to determine which process parameters such as stirrer speed or aeration rate are critical and that within less than 1 h after sampling, compared to 6 days that are necessary to yield a result in form of visible colonies when applying a plating assay. Further, identifying stress parameters in the upscaling to an industrial process is facilitated. Thereby, this FCM method for bioprocess monitoring harbors the potential to pave the way for the first industrial process with *S. acidocaldarius* or related Crenarchaeota.

## Supplementary Information

**Additional file 1:** **Fig. S1.** Fluorescence microscope. Mixture of dead and alive cells of *Sulfolobus acidocaldarius* stained with fluorescein diacetate (FDA) and concanvalin A conjugated with rhodamine and investigated with a Leica DMI 8 fluorescence microscope (Leica Microsystems, Germany). A: Image acquired via the equipped filter 2 (excitation (ex.) 450-490 nm / emission (em.) 500-550 nm). B: Image acquired via the equipped filter 1 (ex. 532-558 nm /em. 570-640 nm). C: overlay of figures A and B showing metabolically active cells as yellow dots (overlay of red and green)

## Data Availability

Raw data that support the findings of this study are available from the corresponding author upon request.

## Abbreviations

AO: Acridine orange
CFUs: Colony forming units
ConA: Concanavalin A
em.: Emission
ex.: Excitation
FCM: Flow cytometry
FDA: Fluorescein diacetate
FSC: Forward scatter
OD_600_: Optical density
PBS: Phosphate buffered saline
SSC: Side scatter
V_FCM_: Specific viability determined via flow cytometry
V_plating_: Specific viability determined via plating assay
