# Supplementary material for: Flow cytometry-based viability staining: an at-line tool for bioprocess monitoring of Sulfolobus acidocaldarius
Source: AMB Express. 2022 Aug 10;12:107. doi: 10.1186/s13568-022-01447-1 (PMC9365904; doi:10.1186/s13568-022-01447-1)
Supplement: Supplementary file 1 — Additional file 1: Fig. S1. Fluorescence microscope. Mixture of dead and alive cells of Sulfolobus acidocaldarius stained with fluorescein diacetate (FDA) and concanvalin A conjugated with rhodamine and investigated with a Leica DMI 8 fluorescence microscope (Leica Microsystems, Germany). A: Image acquired via the equipped filter 2 (excitation (ex.) 450-490 nm / emission (em.) 500-550 nm). B: Image acquired via the equipped filter 1 (ex. 532-558 nm /em. 570-640 nm). C: overlay of figures A and B showing metabolically active cells as yellow dots (overlay of red and green) [file 13568_2022_1447_MOESM1_ESM.docx]

Supplementary Materials


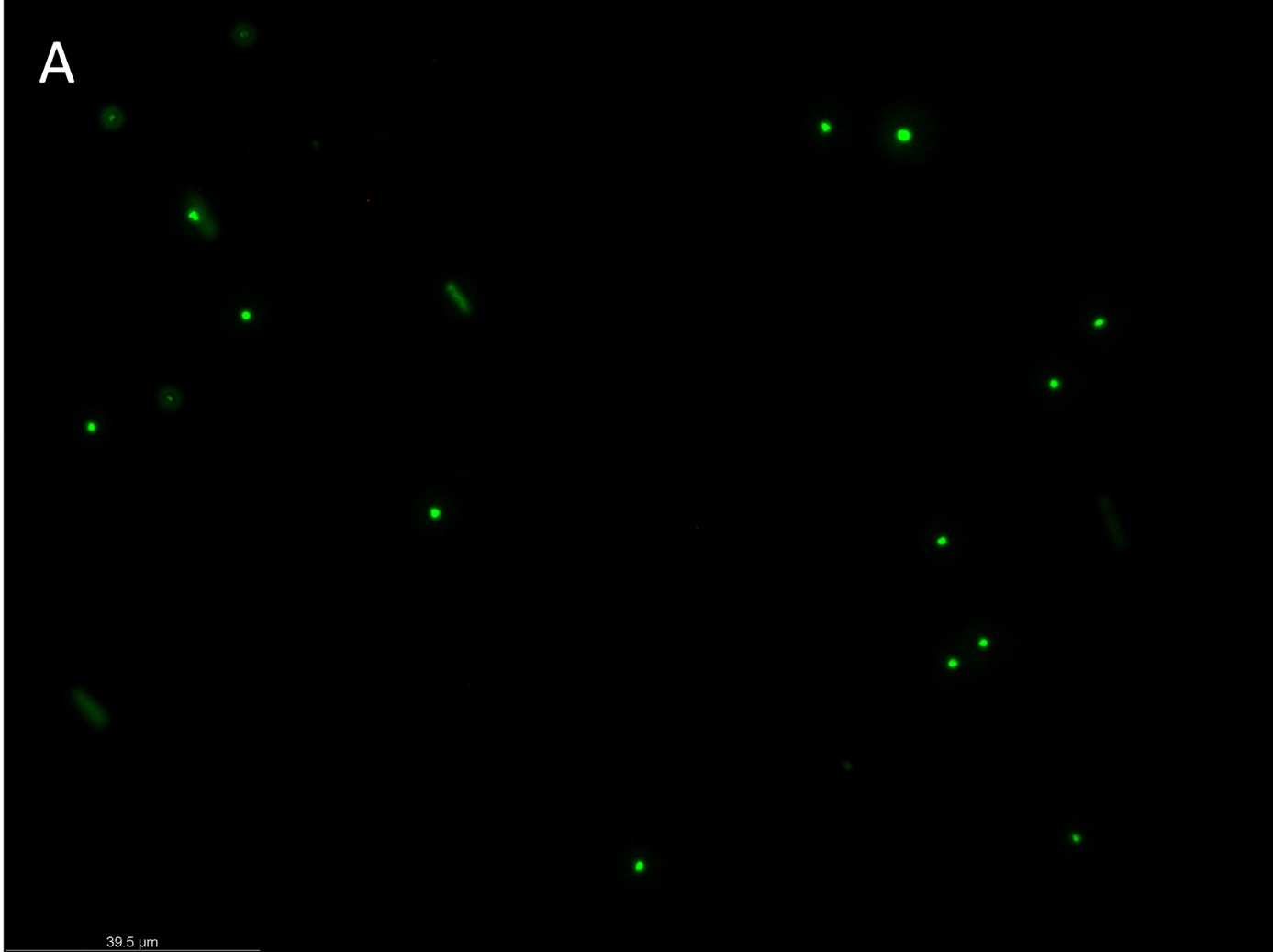


**
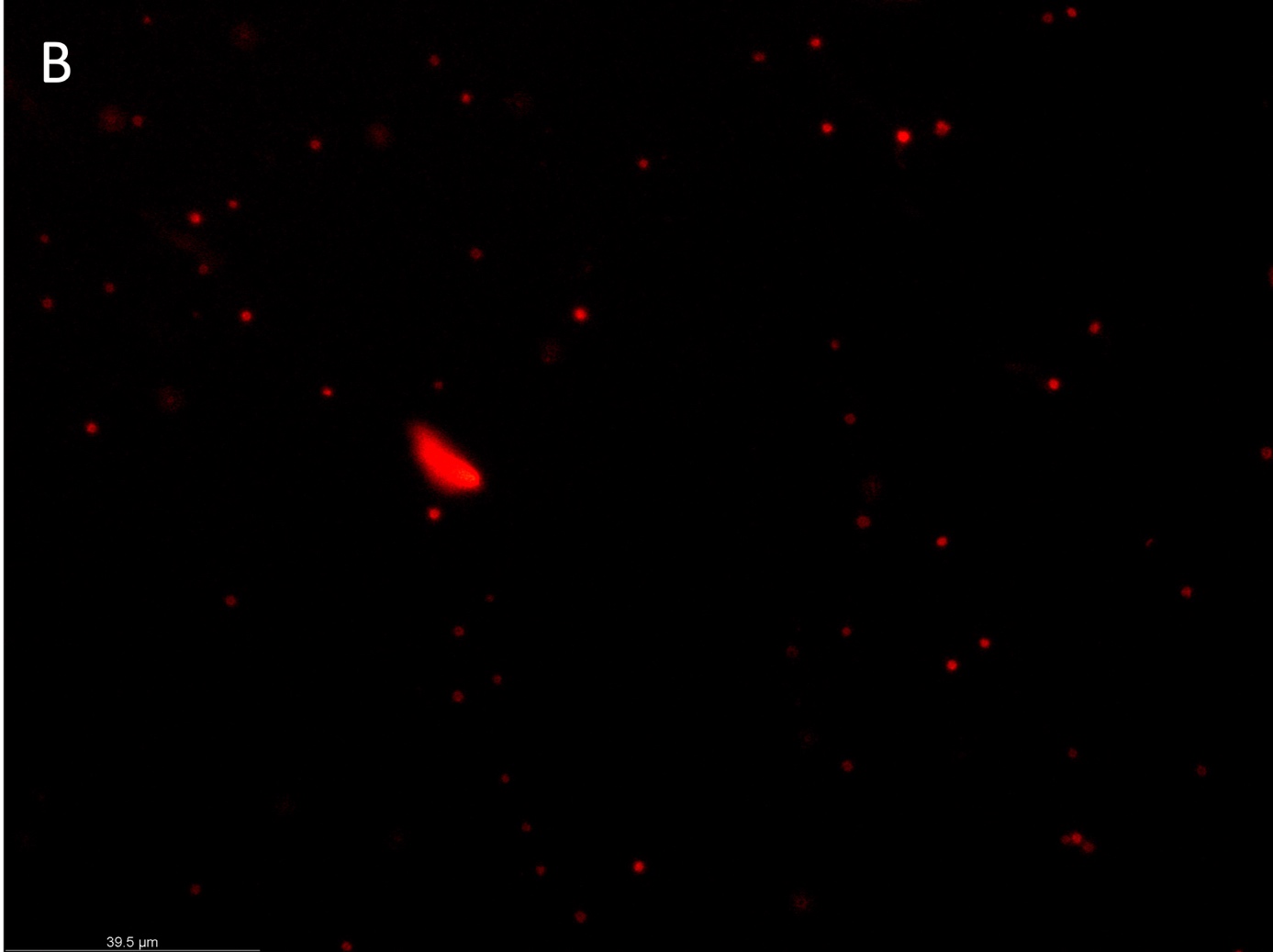
**


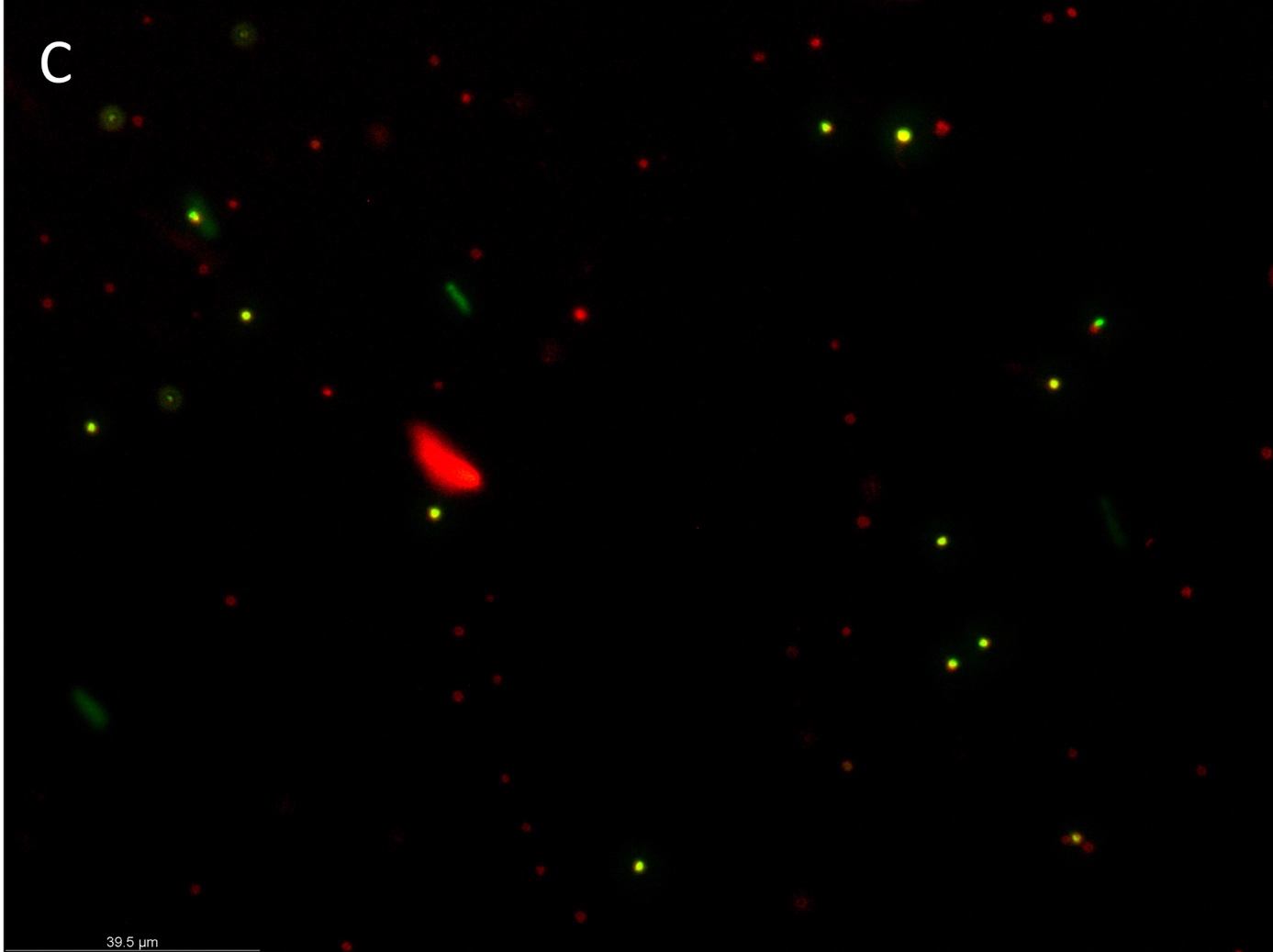


**Fig. S1 Fluorescence microscope:** Mixture of dead and alive cells of Sulfolobus acidocaldarius stained with fluorescein diacetate (FDA) and concanvalin A conjugated with rhodamine and investigated with a Leica DMI 8 fluorescence microscope (Leica Microsystems, Germany). A: Image acquired via the equipped filter 2 (excitation (ex.) 450-490 nm / emission (em.) 500-550 nm). B: Image acquired via the equipped filter 1 (ex. 532-558 nm /em. 570-640 nm). C: overlay of figures A and B showing metabolically active cells as yellow dots (overlay of red and green).
